# Effects of High Larval Density on Wing Shape Deformations of *Culex pipiens* (Culicidae: Diptera) via Geometric Morphometrics

**DOI:** 10.3390/insects16121185

**Published:** 2025-11-21

**Authors:** Seçil Aytekin, Zafer Sakaci, Sengul Talay, Bulent Alten

**Affiliations:** 1 Graduate School of Science and Engineering, Hacettepe University, Beytepe, 06800 Ankara, Türkiye; 2 Department of Biology, Tekirdag Namık Kemal University, Suleymanpasa, 59030 Tekirdağ, Türkiye; zafersakaci@yahoo.com (Z.S.); talay_59@hotmail.com (S.T.); 3 VERG Laboratories, Department of Biology, Faculty of Science, Hacettepe University, Beytepe, 06800 Ankara, Türkiye

**Keywords:** mosquito, landmarks, Elliptic Fourier Analysis, Centroid Size, breaking point

## Abstract

Populations of *Culex pipiens,* which is considered a primary vector of West Nile Virus, are not uniformly shaped, and hence, they are difficult to work on, not only because of the complex structure of the species but also due to the possible deformations caused by several factors like temperature, pH, parasite, and bacterial density. Larval density is another crucial factor. This study summarizes the effects of larval density in *Cx. pipiens* as a model of experimental semi-controlled ecomorphs by two different geometric morphometrics methods. The landmark-based method explains that dimorphism is clearly visualized in both the size and shape of the wings. It also shows that females and males have gradually traceable deformations. When the population reaches a high larval density, which is calculated as approximately 0.5 cm^3^/individual or, in other words, 1 larva/mL in *Cx. pipiens,* it can be considered a breaking point, where deformations in shape are observed through all study periods, indicating that it is effected separately and varies independently from the other factors. The wings become smaller in both sexes as the larval density increases. Similar results are obtained by Elliptic Fourier Analysis, which explains the difference in the contour of the wing, regardless of where the landmarks on the veins are located.

## Simple Summary

This study focuses on analyzing the shape and size of *Cx. pipiens* by using two different geometric morphometrics methods. Although several well-known methods to visualize the high density concept in ecology have been defined, these methods focus on estimating the high number of individuals and do not concentrate on understanding the exact definition where high density begins to cause real statistically visualized ecological stress. Within this context, it is important to understand not only the effects of high larval density but also define the concept with an objective criterion. This way, it would be possible to define the breaking point when the density is high enough to cause deformations in a given population. Our results showed that the size of the wings became gradually smaller as the larval density increased, especially when the larval density reached approximately 128 individuals (1 larva/mL), which can be defined as a very crowded area. The wing vein structure was deformed independent of the wing contour. Since the wing size and shape changed as the larval density differed, taxonomical and phylogenetic studies also need to reconsider the source of the material in use a priori.

## 1. Introduction

A crowd refers to a large number of individuals gathered together in a specific area. It is a collective term that emphasizes the presence and behavior of individuals within a group. The movement and speed of individuals in a crowded population are influenced mostly by physical factors, and a high crowd density can pose several risks [1]. Conversely, “density” defines the concentration of individuals within a given area. It is a quantitative measure that indicates how crowded a space is. Density can be measured using various techniques [2], but there are limitations in defining and standardizing the exact divergence points for high, low, and optimal numbers of individuals in a given population.

A high larval density can lead to physiological stress in animals, increasing susceptibility to infection and impacting body size, development rates, and immune response [3,4,5,6]. For instance, in *Aedes* sp., larval density influences survival, growth rate, and body size, with implications for population regulation and control strategies [7,8]. A high larval density and prolonged food shortage leads to a reduction in the size of adults and increased mortality rate in *Aedes aegypti* [9]. The density-dependent effects at the expansion front of a species can accelerate range expansion by increasing survival and individual growth rates, as well as enhancing investment in dispersal ability [7]. Understanding the effects of larval density on mosquito phenotypes is crucial for vector control strategies. Larger mosquitoes, which can result from lower larval densities, may have higher vectorial capacities, making them more efficient disease vectors [10]. Therefore, controlling larval density through environmental management could be a key strategy in reducing the spread of mosquito-borne diseases.

It is possible to digitally define a “form” and, in this way, conduct comparative statistical analyses on any object. D’Arcy Thompson (On Growth and Form, 1917) was the first to use this approach to compare the structures of different species of organisms and explain these as specific changes in form or deformations by simple graphical expression [11]. Research proceeding with this approach later led to the development of the Old Morphometrics. In the 1960s, Oxnard, one of the founders of this school, studied these forms by plotting the same structures across species on deformation grids, calculating variances and thus enabling statistical analysis of the diversity of phenotypic differences [12]. Although this enabled a certain understanding of structural differences at a specific scale, comparing these deformations with thin plate splines (TPS) only became possible approximately two decades later with the development of modern geometric morphometrics [11,13]. Subsequently, the ability to analyze landmarks of biological structures superimposed on each other by digitizing them on relative warps using General Procrustes Analysis completely transformed the process and made geometric morphometrics applicable in diverse fields such as evolution, ecology, systematics, and taxonomy. This development, which enabled the construction of landmark-based geometric morphometrics methods used today, led to radical changes not only in methodology but also in conceptual and statistical terms. Modern geometric morphometrics, defined as the statistical analysis of shape variation and its covariation with other variables, has been considered a revolution in studies since then [14]. Over the past 30 years, numerous studies have been published in different disciplines, from systematics, anthropology, ecology, evolution, medicine, dentistry, and physiology to archeology and even automotive mechanics [15].

Geometric morphometrics can be used as a very powerful tool for studying the phenotypic variations in mosquitoes caused by any factor [16,17]. It has been used to study the phenotypic differences in mosquito populations under varying larval densities. In *Aedes albopictus*, significant differences in wing Centroid Sizes were observed between different larval densities, although wing shape remained relatively consistent [18]. The effects of larval density on wing morphology also exhibit sexual dimorphism. In *Culex pipiens* and most of other mosquito species, females are typically larger than males, reflecting sexual dimorphism linked to reproductive physiology and energy allocation [10,19,20]. In *Culex pipiens*, high larval density leads to smaller females, while fluctuating temperatures result in larger males [21].

High larval density in mosquito populations may cause several deformations. It has different effects on both sexes and this can be affected in different seasons. In order to define the point where the population’s response to the pressure which causes deformation of the wings becomes evident, in this study, we analyzed and visualized the effect of different larval densities on wing shape and size, also considering overall wing contour, with two different geometric morphometric methods in a *Culex pipiens* controlled population reared at different density levels to make a standardized definition of a breaking point where changes in form occur.

## 2. Materials and Methods

### 2.1. Study Area and Design

All adult *Cx. pipiens* spp. specimens used were obtained from the study conducted by Sakaci et al. [22]. The *Cx. pipiens* populations used in the study were captured in a periurban area, and our study was carried out in Tekirdağ province, in the northwestern part of Türkiye (40°58′ N, 27°30′ E, 40 m), during the period when *Cx. pipiens* laid eggs in the natural environment, in 2021–2022. We used some of the material co-analyzed for the study of Sakaci et al. [22]. We selected this study area because, in this area, it is known that *Cx. pipiens pipiens*, *Cx. pipiens molestus*, and hybrid individuals co-exist. Therefore, after preliminary analysis of the specimens, only specimens of *Cx. pipiens* (s. str.) were used for the analysis.

**Figure 1:**
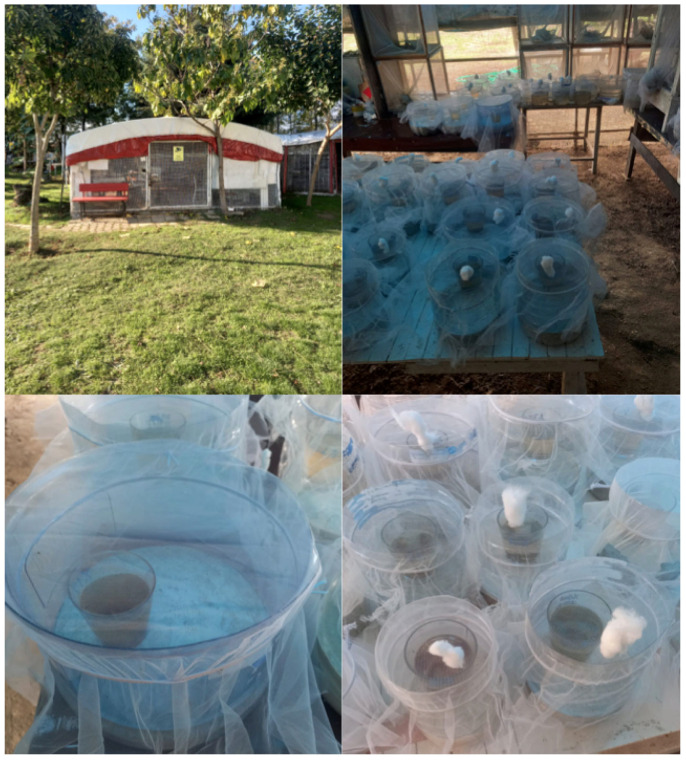
Images of the study area and experimental setup, showing the greenhouse in the field and the containers used to rear larvae.

The study was carried out in a greenhouse mesocosm covered with tarpaulin and wire mesh [22]. Precautions were taken to ensure that the monitored mosquito larvae were not directly affected by rain and sunlight (Figure 1).

Hourly temperature and humidity data were recorded during the whole study process using digital dataloggers (TFA Dostmann KlimaLogg Pro 30.3039-TFA Dostmann GmbH & Co. KG, Wertheim-Reicholzheim, Germany). The larvae used in this study were obtained directly from egg rafts produced by the natural *Culex pipiens* population inhabiting the area surrounding the greenhouse. To collect the eggs, 14 oviposition containers filled with dechlorinated tap water were placed near the experimental site and inspected daily. Additional containers naturally present in the area were also monitored. Collected egg rafts were transferred to incubation trays containing 150 mL of water enriched with finely ground baby fish food. The trays were kept under greenhouse conditions until hatching. Once the eggs hatched, first-instar larvae (L1) that emerged on the same day were pooled to form a homogeneous stock and were subsequently distributed into the experimental containers to establish the different larval density groups. This procedure ensured that all larvae used in the experiment originated from the same natural population, minimizing potential maternal or developmental biases and allowing for consistent comparison across density treatments. PET caps with a size of 6 × 4 cm each and experimental groups were formed on the day the first-instar larvae hatched. Larval densities were determined in six different density groups; the first group contained 8 individuals, the second group had 16 individuals, the third group had 32 individuals, the fourth one had 64 individuals, the fifth one had 128 individuals, and the final one had 256 individuals, corresponding to densities of 0.06, 0.12, 0.25, 0.50, 1.00, and 2.00 larvae per mL, or approximately 10, 5, 2.5, 1.5, 0.5, and 0.25 cm^3^ per larva, respectively. These values were used to describe density levels in both volumetric and numerical terms to ensure cross-comparability with other studies. The water volume was kept constant across all treatments, and daily monitoring ensured that evaporation losses were compensated with freshly added water. This procedure maintained a consistent physical space per larva throughout the experiment, providing a standardized reference for assessing crowding effects. To eliminate potential nutritional bias and ensure that the effects observed were solely density-dependent, a non-food-limiting feeding regime was applied. Baby fish food (commercial powder type) was finely ground and weighed using a precision microbalance according to the estimated per capita larval requirement of approximately 0.09 mg per larva per day, and the feeding regime was designed to maintain a non-food-limiting condition, allowing us to isolate the effects of larval density itself. A constant per capita food amount (~0.09 mg per larva) was chosen based on standard nutritional requirements reported for *Culex* and other species [23,24,25,26]. Evaporative water loss was compensated daily with dechlorinated tap water to maintain consistent water volume across all treatments. Feeding was not performed daily but instead followed an instar-based intermittent schedule. Because larval development time varies with ambient temperature, the timing of feeding events was adjusted accordingly. At two-day intervals, four larvae were randomly selected from each experimental group and examined under a stereomicroscope in liquid medium to determine their developmental instar stages [27]. After the initial feeding on Day 0 (first-instar stage), the next two feedings were performed when larvae reached the early third (L3)- and early fourth (L4)-instar stages, respectively. This corresponded approximately to every 3–4 days in June and July, and every 4–5 days in September. At each feeding event, additional food proportional to the current larval density was supplied (0.09 mg × number of larvae × feeding fraction), ensuring that the nutritional conditions were standardized across all density groups. To avoid disrupting the natural dynamics of larval crowding and to better simulate ecological conditions occurring in nature, dead larvae were not removed during the experiment. This design maintained comparable energy availability while reflecting natural fluctuations in resource use. The study was performed across 3 different seasonal periods. The first group was established in June, as it was the first month when intensive egg laying began and possible larval crowding could develop in containers in their natural habitat. The second group was established in July, when temperatures peaked, and the third group was established in September, when intensive egg laying was observed. The average ambient temperatures measured during the trails were 23.8 ± 1.6 °C in June, 25.8 ± 1.2 °C in July, and 20.2 ± 3.1 °C in September (Table 1). The general data of temperature reflected the typical transition from early-summer to late-autumn conditions in the region. The mosquitoes emerging from each group were collected and transferred to −20 °C Deepfreeze. All specimens were then reanalyzed under a microscope and separated for further analyses.

### 2.2. Preparation of the Wings

The wings were removed from the thorax and transferred to the digital environment using a Leica MZ-7.5 stereoscopic dissection microscope and a DC-300 digital camera at 16× magnification (Leica, Antlab Lab. Equipments, Antalya, Türkiye). The wings were kept in distilled water, and their scales were mechanically shed with the help of a dissection needle, before being photographed on slides [28]. All analyses of females and males were performed separately. In all examples, the right and left wings are positioned in accordance with their natural appearance. In order to avoid repeated data in the comparison of the effect of larval density between groups, only the right wings were used. Each image of the right wing included a scale bar (1 mm) and was assigned an identification number to minimize errors. Device validations were performed in order to increase millimetric precision.

### 2.3. Digitization and Validation of Data

To estimate the precision of the digitization of each wing element, the measurement error was evaluated using the repeatability index [29]. The evaluation was performed using two sets of images from the same sample twice and the same experimenter digitized the images, and the resulting repeatability was calculated [15,30]. One experimenter performed all analyses, measurements, and illustrations, and to reduce the risk of measurement error, absolute precision and accuracy values were calculated in each analysis to describe and capture the wing shape [31,32]. In order to avoid graphical noise and statistical bias, 15 individuals across both sexes were randomly chosen from each group for analysis of all analyzed groups.

### 2.4. Landmark-Based Geometric Morphometrics

**Figure 2:**
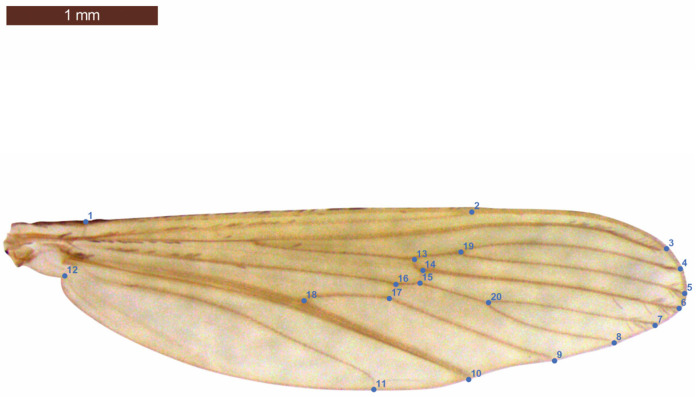
The 20 landmarks used in the study (from the right wings of *Culex pipiens*). Bar indicates 1 mm.

An optimum landmark design scheme was prepared from archived photographs, tested statistically, and 20 landmarks were included in the analysis [33,34]. After obtaining TPSDig [35] data, General Procrustes Analysis was performed with photographs of the 20 landmarks used in the wings (Figure 2). Then, the unit weight center (Centroid Size), which is a measure of the isometric estimation of the wing size and is known as the square root of the square of the distances of all landmarks to the center mean, was scaled, and wing sizes were calculated together for each individual. The mm unit was used for scale in the calculation stage (mm ± SD) [36,37]. The size results were visualized via 2D graphics, and the parametric data were compared by one-way ANOVA and Tukey’s post hoc test [38]. For the analysis of landmarks; TPS Series (TPSUtil, TPSDig, TPSRelW) was used for the analysis [35,39], and graphics were prepared in MorphoJ Ver. 1.08.02 [40] and PAST 4.0 [41,42]. All statistical tests were performed in PAST Ver. 2025/5.2 [43] and MINITAB© Ver. 22.4.0 (Minitab LLC, State College, PA, USA, 2025). The significance threshold for all statistical analyses was *p* ≤ 0.05. All results were derived from Principal Component Analysis, with the first 3 PCs visualized via 3D modeling. Each graph contains the shape deformation of the wings through the first 3 axes, along with relative warps, shown to illustrate the deformation from the mean shape. Each landmark on the wing is linked with its natural appearance of the veins to provide a better visualization. A Canonical Variate Analysis was also performed to explain the variance among groups, and a graph explaining the first two canonical variates is also given [11,14]. All analyses were performed for 3 periods (June, July, and September) within 6 different density groups (with 8, 16, 32, 64, 128, and 256 individuals in each container), and the results were compared. The profile drawings of the data sample means (only size) and variances (both size and shape) and their 95% resampling values were calculated as the optimal number for explanatory power. Confidence intervals (0.025 and 0.975 percentiles; 1.000 Bootstrap) were used, and the sample sizes tested with this model were found to be appropriate; statistical analyses were performed with an ANOVA [17]. Box’s M statistics are provided, along with significance values based on chi-square approximation, which is a sensitive indicator of inequality of the covariance matrices for two or more multivariate samples marked with different columns assumed by Multivariate Analysis of Variance (MANOVA), which is a multivariate version of the univariate ANOVA, testing whether all mentioned 6 density groups have the same multivariate mean. Wilk’s Lambda and associated Rao’s *F* values are given for each group’s explanatory power in % values in the graphs derived from General Procrustes Analysis of the obtained data [43]. The pairwise comparisons (post hoc) proceeded if the MANOVA showed significant overall difference among groups by pairwise Hotelling’s tests, and only values *p* ≤ 0.05 are mentioned in the text. There were no missing data, so no column average substitutions were used, and an overall MANOVA test of multivariate regression significance for all density groups was also tested (not given) to follow the nature and possible origin of the variations.

### 2.5. Outline-Based Geometric Morphometrics

The Elliptic Fourier Outline method was used, and Bitmap files were imported into the SHAPE Ver. 1.3, which generated elliptical Fourier descriptors via creation of a chain code to analyze the wing contour area [44]. This chain code process uses the binarized picture that the software produces and encircles the object in question counter-clockwise, creating an x and y sequence of ordered points beginning at an arbitrary point along the object. SHAPE created elliptical Fourier descriptors (EFDs) based on this chain code [45]. Twenty harmonics accurately represented artifact shape when taking into consideration the resolution of photographic digitization, as well as the flint knapping process itself. Graphics obtained from the calculated PCs based on EFDs were created in R Ver. 4.3.3. [46]. The first two Principal Component scores were visualized using a series of box plots and scatter plots to assess whether any of these Principal Components create the same distribution over time. A *t*-test was performed on the separated discriminant function scores for groups [47].

## 3. Results

The measurement error in digitizing the wing elements was calculated to be low, ranging from 2% to 4% for different shapes. Correspondingly, the repeatability scores were very high, with values of 99 for the wing contour and landmark data, which indicates that the digitizing process in our study was highly accurate, with measurement errors remaining low across all examined elements.

### 3.1. Landmark-Based Geometric Morphometrics

#### 3.1.1. Two-Dimensional Principal Component Analysis

**Figure 3:**
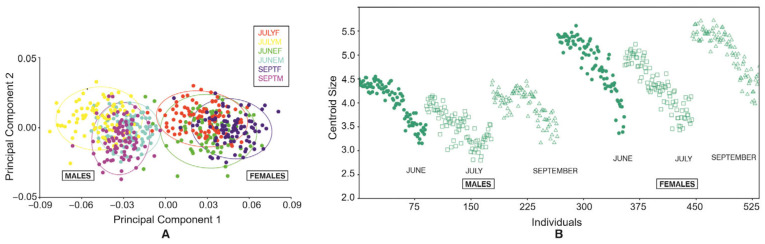
(**A**) Principal Component Analysis of *Culex pipiens* examined in the study explaining the sexual dimorphism in landmark data on the first two PCs. Colors indicate different seasonal periods. Each point represents one individual. (**B**) Distribution of the Centroid Sizes among the same periods.

The superimposition of the mean shapes in each wing element highlighted shape differences among sexes in all periods (June, July, and September). Notably, the first two Principal Components explained the 96.15% of the total variation, which suggests a clear size difference between females and males (Figure 3A), where females have clearly bigger wings (*p* < 0.005 ANOVA F = 134.27) among all periods (Figure 3B).

#### 3.1.2. Three-Dimensional Principal Component Analysis

**Figure 4:**
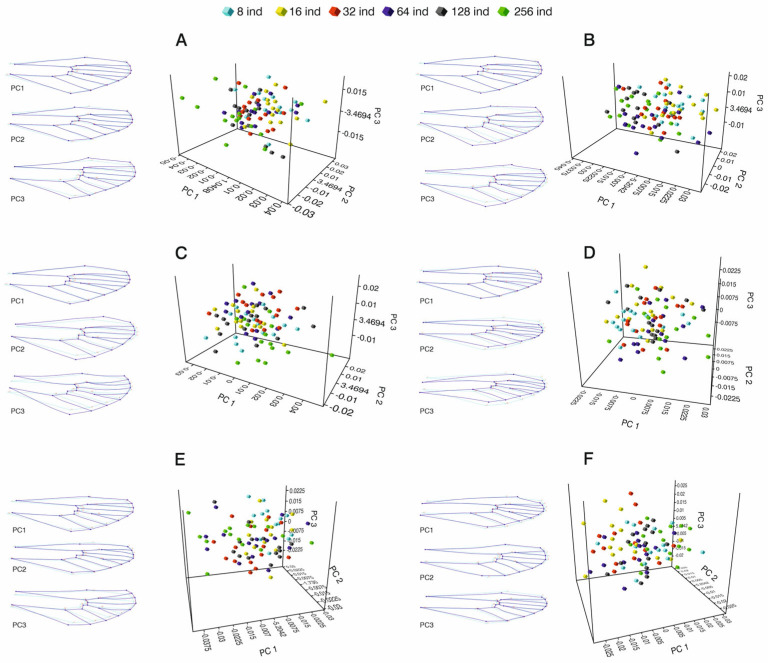
Distribution of wing shape across right wings from *Culex pipiens*, with comparison of mean shape configurations for each sex and seasonal period. Light color indicates mean shape, whereas dark color indicates the deformation for each PC (PC1, PC2, and PC3). In the 3D PCA graphs, each color represents different larval densities. Each 3D graph has been rotated to better visualize the groupings. (**A**) Females (June); (**B**) females (July); (**C**) females (September); (**D**) males (June); (**E**) males (July); and (**F**) males (September).

The shape and size of each wing were analyzed separately for each period and differently for males and females. For June; regarding the distribution of differences in wing structure in the density effect groups comprising 8, 16, 32, 64, 128, and 256 individuals, the first three Principal Components explained 28.10%, 18.59%, and 12.45% of the total variance, respectively. In females, the first three PCs explained 59.14% of the total variance in the examined data (Figure 4A) (*p* < 0.005 MANOVA F = 745). For July; regarding the distribution of the differences in wing structure in the density effect groups comprising 8, 16, 32, 64, 128, and 256 individuals, the first three Principal Components explained 31.60%, 12.87%, and 10.34% of the total variance, respectively, with a cumulative explanation power of 54.81% (Figure 4B) (*p* < 0.005 MANOVA F = 722). Similarly, for September; the first three PCs explained 25.31%, 16.63%, and 12.27% of the total variance in females, respectively (Figure 4C), with an explanatory power of 54.21% (*p* < 0.005 MANOVA F = 643). Similar results were obtained for males. For June, the first three PCs explain 19.28%, 15.80%, and 13.63% of the variance (thus explaining 48.71% of the total variance in the examined data) (Figure 4D) (*p* < 0.005 MANOVA F = 632). For July, the first three PCs explain 27.93%, 14.88%, and 14.24% of the variance, resulting in a cumulative explanation power of 57.05% (Figure 4E). Finally, for September, regarding the distribution of differences in wing structure in the density effect groups comprising 8, 16, 32, 64, 128, and 256 individuals, the first three Principal Components explain 23.51%, 14.92%, and 12.24% of the variance, respectively (Figure 4F), thus explaining 50.68% of the total variance in the examined data. In all seasons, deformation of the wings mostly occurred in landmarks located in the basal and apical parts. However, the males formed a more tapered shape towards the apical part (Figure 4D,E); females showed expansion towards the median compared to the mean shape of the landmark configurations for each PC. The bending energy in the warps clearly show the shape deformation in all groups, thus visualizing the shape changes explained by the second and third Principal Components, with the size changes explained by the first PC.

#### 3.1.3. Canonical Variate Analysis (CVA)

**Figure 5:**
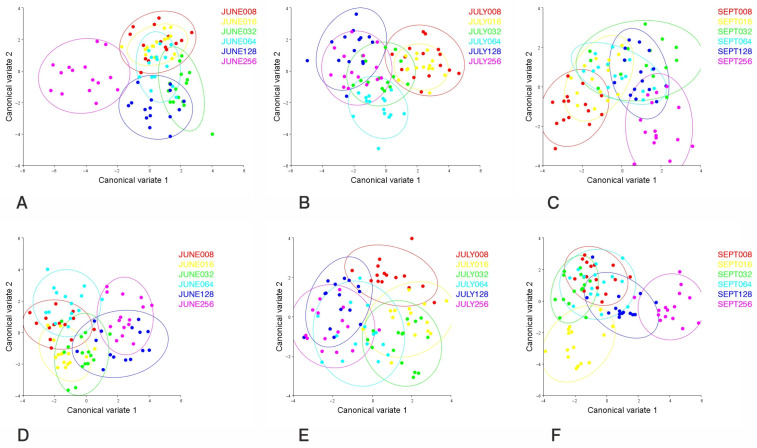
Canonical Variate Analysis of *Culex pipiens* examined in the study, explaining different density groups using 20 landmarks. Colors indicate different larval densities. Each point represents one individual. Groupings in circles formed by 95% intervals. (**A**) Females (June); (**B**) females (July); (**C**) females (September); (**D**) males (June); (**E**) males (July); and (**F**) males (September).

To better visualize the pattern of grouping among density groups Canonical Variate Analysis (CVA) was also performed, showing that the density groups comprising 128 and 256 individuals clearly segregated into different clusters compared to the rest in females in June (Figure 5A), with the variation among groups scaled by the inverse of the within-group variation of 76.34% (*p* < 0.005 MANOVA F = 318). The density groups segregated clearly into different clusters in July (Figure 5B), with variation among groups scaled by the inverse of the within-group variation of 70.87% (*p* < 0.005 MANOVA F = 318), showing a pattern similar to that of June and very similar to that of September (within-group variation of 65.60%) (*p* < 0.005 MANOVA F = 511) (Figure 5C). In males, the situations were very similar for all study periods. In June, all groups also separated clearly, as in females (Figure 5D), with a cumulative variation of 66.62% (*p* < 0.005 MANOVA F = 216). Similar results were observed in July and September, as the first two Canonical Variates explained 59.66% of the total variation (*p* < 0.005 MANOVA F = 216) (Figure 5E) in July and 74.44% of the total variation (*p* < 0.005 MANOVA F = 416) in September (Figure 5F).

#### 3.1.4. Centroid Sizes

**Figure 6:**
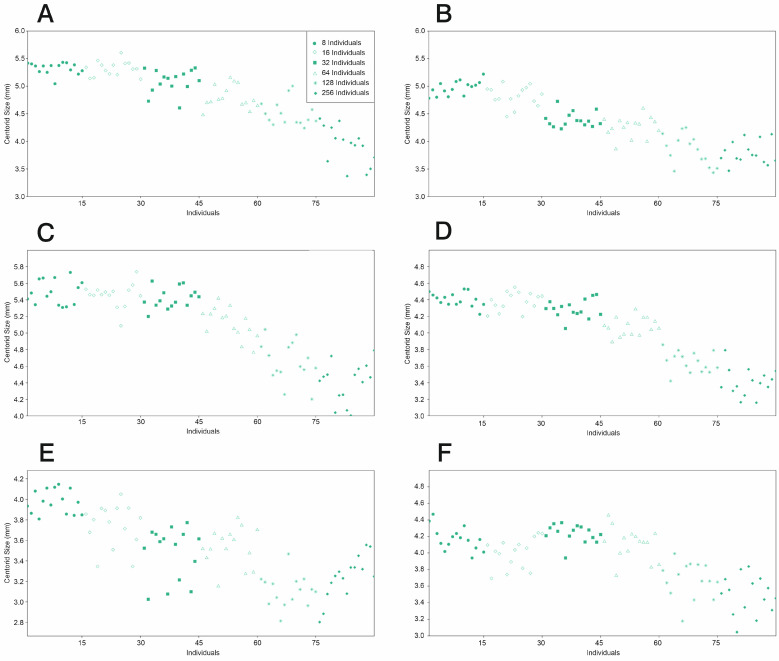
Distribution of Centroid Sizes of *Culex pipiens* examined in the study using 20 landmarks. Each figure represents one individual. Different geometric figures represent density groups. (**A**) Females (June), (**B**) females (July), (**C**) females (September), (**D**) males (June), (**E**) males (July), and (**F**) males (September).

The differences in wing structure in all density effect groups in June had an effect on the variability of the landmarks, as shown by the Centroid Size values, a typical indicator of size. It can be observed that the wings become gradually smaller as the density increases in females (Figure 6A) (*p* < 0.005 ANOVA F = 93.81), and separation occurred in all but the 8-individual and 16-individual groups, which overlapped. Very similar results were obtained in July and September. It can be observed that the wings become gradually smaller as the density increases (Figure 6B) (*p* < 0.005 ANOVA F = 96.55), as in June, but in July, the female groups had pairings in terms of wing size, as the 8–16, 32–64, and 128–256 groups had similar Centroid Sizes. In September, it can be observed that the wings gradually became smaller as the density increased in females (Figure 6C) (*p* < 0.005 ANOVA F = 93.47), and the first three groups—containing 8, 16, and 32 individuals, respectively—had similar Centroid Sizes, whereas the others did not, like in June. A very similar pattern was observed in males, with some slightly different nuances, like in June (Figure 6D) (*p* < 0.005 ANOVA F = 40.33). In July, the wing size groupings were similar to those in females, as the 8–16, 32–64, and 128–256 groups had similar Centroid Size values. In July, the first three groups (containing 8, 16, and 32 individuals, respectively) had size similarities, whereas the other groups had clearly different values (Figure 6E) (*p* < 0.005 ANOVA F = 180.90), as in females. In September, the pattern was also similar to that of the females (*p* < 0.005 ANOVA F = 40.04), being very similar to the results obtained in June, in which the 8–16- and 32-individual were similar, and the 128–256 groups were not (Figure 6F).

### 3.2. Outline-Based Geometric Morphometric-Based Elliptic Fourier Analysis

**Figure 7:**
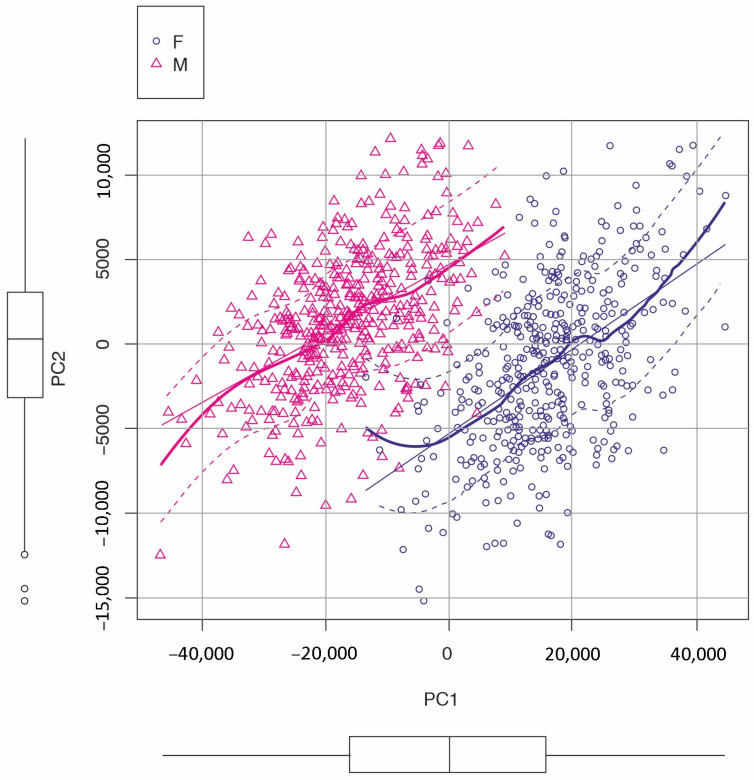
Elliptic Fourier Analysis of right wing contours in *Culex pipiens* reared in different seasons and at densities, with the first 2 PCs showing sexual dimorphism. Each figure represents one individual. Boxplots depict the variation in PCs. Each sex is represented by a different color and geometric shape.

Females and males differed drastically, according to the Elliptic Fourier Analysis, in terms of the landmark data defining the whole wing contour (*p* < 0.005 *t*-test), with the first two Principal Components explaining 86.20% of the total variance. Defining the median of the wing, it is slightly more elliptical in shape in females compared to males. These data are similar to the landmark configuration data (Figure 7), which indicates a similar dimorphism.

## 4. Discussion

Mosquitoes, belonging to the Culicidae family, are considered one of the most prevalent insects responsible for disease transmission in humans. *Cx. pipiens* is a complex species of great research interest, being considered as the primary vectors of West Nile Virus (WNV) [48,49]. They play a role in the transmission of WNV to birds and mammals, including humans, because they feed on both avian and mammalian hosts [50]. Studies based on molecular methods have confirmed that members of the *Cx. pipiens* complex are widely distributed in different regions of Türkiye [51,52]. The *Culex pipiens* complex is known to be widespread across Türkiye; however, comprehensive studies on its geographical distribution and species composition within the country remain lacking. Existing entomological surveys on mosquito fauna indicate the presence of *Cx. pipiens pipiens*, *Cx. pipiens molestus*, *Cx. quinquefasciatus*, and their hybrids. Nonetheless, these records are currently confined to the eastern, central, and Aegean regions of Türkiye [53]. Our study was conducted under semi-field conditions that closely reflected the natural thermal and environmental conditions of the study site. The greenhouse mesocosm was located within the natural habitat range of *Culex pipiens* populations in Tekirdağ province, northwestern Türkiye. The structure was uninsulated, without artificial lighting or temperature control, allowing natural fluctuations in temperature and humidity to mirror those occurring in the surrounding peri-urban environment. The local climate in this region is characterized by the interaction of Mediterranean and continental influences, resulting in marked diurnal and seasonal thermal variation. These environmental characteristics are consistent with the conditions under which *Cx. pipiens* naturally develops during the breeding season. Consequently, the experimental setup ensured that larvae were exposed to realistic environmental stressors, such as temperature variation and humidity changes, thereby enabling ecologically meaningful interpretation of density-dependent effects.

Geometric morphometrics is clearly a very powerful tool for evaluating correlations between shape and environmental or genetic variables, especially in mosquitoes and other insects, showing two-dimensional and phenotypic changes such as wings [16,17]. Apart from inter-species differences, it is explanatory for many different phenomena, such as plasticity; asymmetry; general physical, chemical, and biological factors; and, in particular, ecological factors [54,55]. It is known that the phenotypic change gradient in mosquitoes is affected by various stress conditions, such as temperature in the areas where eggs are laid [56], pH, parasite and bacterial density [57], and the crowding effect [58], which can be measured morphometrically, and these factors can strongly affect adult fitness in *Culex pipiens*. Elevated temperatures above approximately 26–28 °C are known to accelerate larval metabolism and reduce the size and energy reserves of emerging adults, thereby decreasing their survival, dispersal capacity, and reproductive potential. Conversely, suboptimal or fluctuating pH levels and increased organic matter concentration can impose physiological stress during the larval stage, altering osmotic balance and reducing developmental efficiency. In our semi-field setting, natural variations in temperature and water chemistry likely interacted with density-dependent stress, amplifying its impact on adult traits. Such interactions may lead to smaller, less viable adults under high-temperature or low-humidity conditions. Therefore, environmental parameters act as secondary but synergistic factors modulating the effect of larval density on the overall fitness and population dynamics of *Cx. pipiens*. Collectively, these findings highlight a multifactorial interaction in which larval crowding, temperature, and water chemistry act in concert to shape adult morphology and fitness. Understanding this interplay is essential for predicting the ecological resilience and vectorial capacity of *Culex pipiens* populations under fluctuating environmental and climatic conditions. It has been shown that crowding causes physiological and structural changes in mosquitoes, as in many other living organisms. Studies have shown that most of these effects are often negative, including decreased egg production [59], a shortened lifespan, and reduced wing length [60]; significantly increased mortality [58], parasite transmission by contact; toxin accumulation due waste; chemical signals released by larvae and nutritional differences due to food partitioning [61]; and significant differences in developmental times [62]. Also affected are the dispersal range, energy optimization during flight, and even elevation and flight performance [63].

In recent years, the general trend in geometric morphometrics has been to conduct two different phenotypic examinations of form and shape [15]. The wing size and shape of mosquitoes can exhibit sexual dimorphism, with wings being a potential indicator of sex in mosquitoes. There has been limited direct information regarding the differences in wing venation between male and female *Cx. pipiens* mosquitoes. Significant sexual dimorphism in wing morphology has been observed in other mosquito species, like *Aedes fluviatilis*, indicating evolutionary pressures leading to distinct wing characteristics between genders [64]. Wing length has been used as a proxy for body size in studies of *Cx. pipiens*, with larger females generally having longer wings compared to smaller females [65,66]. This suggests that wing length may vary between genders, although specific venation patterns have not been detailed. Our finding that females exhibit larger wing sizes than males under all density and temperature regimes is consistent with previous studies on *Cx. pipiens* and other mosquito species. Similar sexual size dimorphism has been observed under varying environmental conditions, where females generally invest more in somatic growth due to the longer larval development time and greater nutrient accumulation [67,68,69]. The physiological basis for this difference is linked to sex-specific metabolic demands and protandry, which allows males to emerge earlier but at a smaller body size. In our study, increasing larval density and higher rearing temperatures (notably during July) both contributed to a proportional reduction in wing size in both sexes, yet the relative size gap between females and males persisted, supporting the idea that sexual dimorphism is robust to environmental stress. Similar patterns were reported in *Anopheles gambiae*, *Aedes aegypti*, and *Ae. albopictus*, where crowding and thermal stress decreased body size but maintained the female-biased dimorphism [70,71,72]. These consistent results suggest that sexual size dimorphism in *Cx. pipiens* reflects a stable adaptive feature rather than a plastic response, even under variable density and temperature conditions. Our results showed very clear size and form changes in the wing venation patterns in each study group, indicating different larval densities. Females and males are found to be different but have gradually traceable deformations in wing shape and size. As female *Cx. pipiens* have been found to have longer flight capabilities and faster flight velocities compared to males, these differences may be influenced by differences in wing structure and venation [73]. The difference in wing beat frequency is likely related to variations in wing venation and structure [74] and could possibly explain changes in flight range and energy consumption during flight. Larger and deformed wings likely need more energy during active flight, thus causing unpredictable fluctuations in the vector capacity of the species. All of the results of our study, across the different density groups and both sexes, seem to support the conclusion that wing shape is evolutionarily more stable than size [75]. After blood feeding, mosquitoes exhibit significant increases in stroke amplitude, mid-down stroke duration, velocity, and flip angles, which may be related to changes in wing venation and aerodynamic characteristics, considering their blood-feeding behavior [76,77,78,79].

We have also reached to a similar conclusion after analyzing both sexes separately, as they showed the same response to high-density stress. Our results also showed that the phenomenon of larval density affects the shape of the wing, and by means of the landmarks, this is a traceable fact for *Cx. pipiens*. The results of this study also reflect the strong influence of temperature on the morphometric traits of *Culex pipiens*. The three experimental periods exhibited distinct thermal regimes (mean ± SD: 23.8 ± 1.6 °C in June, 25.8 ± 1.2 °C in July, and 20.2 ± 3.1 °C in September). These variations appear to modulate the effect of larval density on adult wing size and shape. High summer temperatures, particularly during July, likely accelerated larval metabolism and shortened development time, resulting in smaller adult wings due to physiological stress. In contrast, cooler September temperatures prolonged larval development and allowed for more uniform growth, partly mitigating the negative impact of crowding. This interaction suggests that temperature acts as a secondary but significant covariate influencing the density-dependent morphological response in *Cx. pipiens* populations. Both size and shape deformations seemed to occur gradually in each density group. When the population reaches high larval density, which is calculated as approximately 128 individuals (=0.5 cm^3^/individual—1 Larva/mL) in *Cx. pipiens*, deformations in shape especially affect the apical, median, and basal parts of the wing, which have a key role in wing flapping motions. This finding was observed in all study periods, indicating that effects are separate and vary independently from other factors like temperature, age, and/or nutrition level [80]. This level of larval density seems to be enough to define the point at which the population has reached the maximum number of individuals to carry in per cm^3^. This can be considered as a breaking point for changes in the form and size in *Cx. pipiens*. These results will be helpful for developing vector control strategies and could aid several future ecological-related studies on *Culex spp*., as well as taxonomical research focusing on signals in different mosquito species. Defining an approximate breaking point in laboratory studies and carefully planning (a priori) the number of individuals in each container would help to reduce possible biased results in any ecological study focusing on several factors other than density itself.

Analysis of Fourier harmonics, also known as Elliptical Fourier Analysis (or EFA), better solves the problem of complicated shapes [45,81,82]. EFA measures and mathematically describes the difference between the first ellipse and the original shape [83,84]. We visualized our EFA of the right wings of both sexes, and these data gave exactly the same segregation of the landmark data. In comparison with landmark and semi-landmark geometric morphometric techniques, EFA also removes the requirement for equally spaced intervals along the outline, so it provides more information about shape deformations. It is shown in our results that the contour of the wings is dimorphic. The use of Elliptic Fourier Analysis in this study provided a quantitative evaluation of wing contour variation that complements the landmark-based geometric morphometrics approach. Similar applications of EFA in *Culex pipiens* and related species (e.g., *Cx. quinquefasciatus* and *Ae. albopictus*) have demonstrated that this technique effectively captures subtle changes in complex wing outlines that may not be evident from landmark data alone [85,86,87]. Our results showed that while larval density significantly influenced the venation pattern, it did not markedly alter the external wing contour. This pattern agrees with previous findings suggesting that the wing membrane is less sensitive to environmental stressors than the venation network, which reflects more direct developmental responses to physiological stress. Such stability of the wing outline under crowding stress likely represents an evolutionary constraint that maintains aerodynamic efficiency. Temperature variation during the larval development periods may have contributed to these results by modulating growth rates rather than altering the overall wing shape. Comparable observations were made by Lyimo et al. (1992) and Couret et al. (2014), who reported that temperature and crowding interact to affect body size but not necessarily contour geometry [61,70]. Hence, the absence of significant contour deformation under different densities and thermal regimes in our study is consistent with previous reports and highlights the robustness of the EFA approach for differentiating between structural and surface-level morphological responses. Future studies should focus on a combination of fluctuating asymmetries and the genetic basis of deformations to gain insights into wing structure and egg production under full controlled laboratory studies.

## Figures and Tables

**Table 1:** Summary of temperature data recorded in the experimental periods: data for June, July, and September, including the minimum (Tmin), maximum (Tmax), and mean (Tmean) values of daily ambient temperature recorded inside the mesocosm greenhouse (°C).

Period	Tmin (°C ± SD)	Tmax (°C ± SD)	Tmean (°C ± SD)
June	18.1 ± 2.1 (14.6–23.4)	29.7 ± 2.1 (26.4–33.6)	23.8 ± 1.6 (21.2–27.6)
July	20.3 ± 1.5 (17.3–23.6)	31.8 ± 1.9 (27.9–35.1)	25.8 ± 1.2 (23.6–27.8)
September	14.7 ± 3.8 (7.9–21.8)	26.1 ± 2.5 (21.2–31.3)	20.2 ± 3.1 (14.9–26.1)

## Data Availability

The original contributions presented in this study are included in the article. Further inquiries can be directed to the corresponding author.

## Abbreviations

The following abbreviations are used in this manuscript:

ANOVA	Analysis of Variance
mm	Millimeter
PCA	Principal Component Analysis
